# Supplementary material for: Agreement ℜ of Four Analytical Methods Applied to Pb in Soils from the Small City of St. John’s, Newfoundland, Canada
Source: Int J Environ Res Public Health. 2021 Sep 18;18(18):9863. doi: 10.3390/ijerph18189863 (PMC8467290; doi:10.3390/ijerph18189863)
Supplement: Supplementary file 1 [file ijerph-18-09863-s001.zip › ijerph-1360267-supplementary.pdf]

Supplemental Materials

Agreement of Four Analytical Methods Applied to Pb in Soils from the Small City of St. John's, Newfoundland, Canada.

By Christopher R. Gonzales, Anna A. Paltseva, Trevor Bell, Eric T. Powell, and Howard W. Mielke

There are two sections to the supplemental material, six graphs showing paired LAD regressions between methods and the methods dataset (N=96) of the results for the methods.

Section S1. Supplemental Figure of Pairwise LAD Regressions of the Analysis Results

The supplemental figure below shows the pairwise graphs of the data for LAD regressions between the different methods. The LAD regression minimizes the sum of absolute values of errors and estimates the conditional median. The best fit ($R^1 = 0.849$) is for 1M nitric acid vs. RBALP method compared to other methods. Note the deviations of the data points and the LAD regression line (dotted lines) from the lines of equality (solid lines). Also note that the LAD regression coefficients of determination for all comparisons are $R^1 > 0.789$. Two methods that measure the same variable should have good correlation and good regression. However, correlation and regression address linear associations between variables and are not equivalent to agreement. The pairwise graphs differ from Figure S1 in the main manuscript. For example, the regression results can only be shown by pairwise graphs whereas Figure S1 illustrates all the individual results (N=96 x 4) for each of the methods in a single graph.

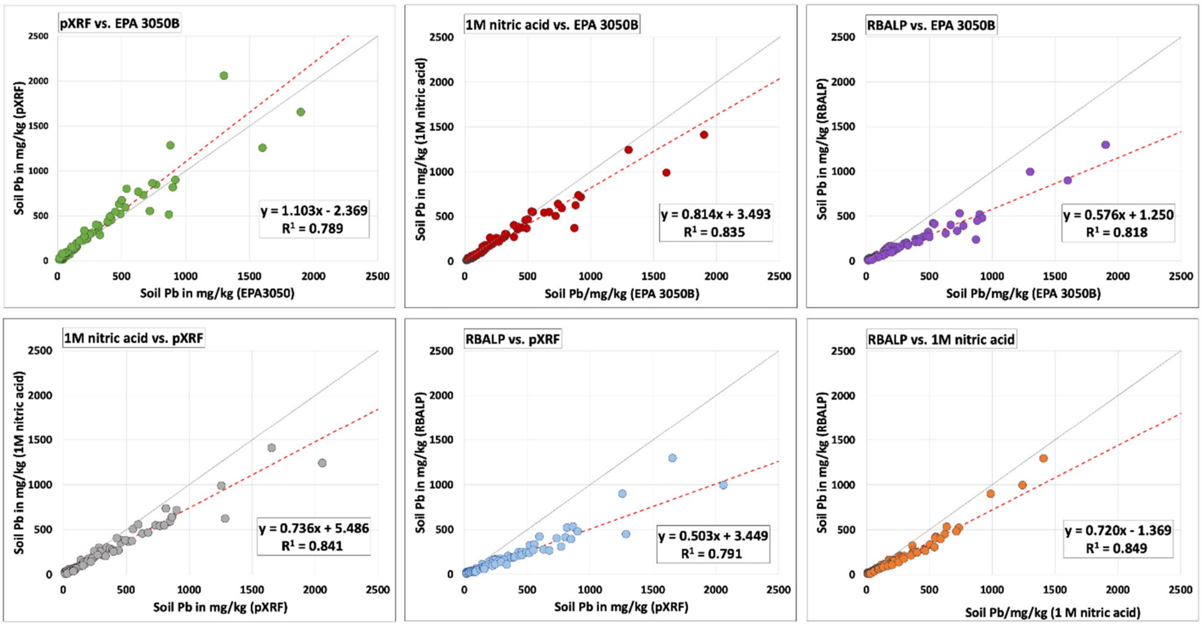

Section S2. Soil Pb Results Obtained from 96 Soil Samples from Four Methods Ranked by pXRF Results

These results from the four methods are graphed in Figure S1.

Soil Pb (mg/kg) by 4 methods					
ID	pXRF Rank	pXRF	EPA 3050B	1M nitric	RBALP
10	1	2058	1300	1242	995
6	2	1654	1900	1410	1295
88	3	1284	880	623	449
49	4	1255	1600	989	899
78	5	900	920	715	479
16	6	862	740	640	532
69	7	847	770	590	393
22	8	815	900	736	520
86	9	801	540	548	412
61	10	767	630	541	306
3	11	732	670	549	401
2	12	673	500	469	265
7	13	629	480	458	276
87	14	595	530	555	420
4	15	553	720	505	334
82	16	545	450	374	268
81	17	519	490	367	323
65	18	514	870	368	239
43	19	492	410	381	246
12	20	455	420	355	212
79	21	454	420	384	247
70	22	437	390	270	210
41	23	427	390	404	245
62	24	398	300	256	194
76	25	390	320	274	188
73	26	370	320	290	188
44	27	346	320	303	206
46	28	335	210	205	108
1	29	311	270	217	160
90	30	292	250	258	138
32	31	285	330	300	176
36	32	272	210	220	150
94	33	246	200	261	168

53	34	243	220	189	113
14	35	242	230	207	158
17	36	229	180	179	163
52	37	222	200	172	95
33	38	220	170	145	118
5	39	200	160	178	145
59	40	194	150	147	109
40	41	172	150	120	106
18	42	163	150	133	81
91	43	156	110	100	59
51	44	151	150	118	74
31	45	142	130	92	75
72	46	140	140	161	115
75	47	130	120	92	66
93	48	124	130	92	58
74	49	102	78	66	48
8	50	99	78	67	46
56	51	93	81	75	67
45	52	88	58	44	31
71	53	85	65	54	36
96	54	85	63	50	43
23	55	83	64	63	35
20	56	81	38	36	25
54	57	79	71	61	45
50	58	72	95	65	41
85	59	68	58	53	35
95	60	62	52	58	39
26	61	57	40	38	28
42	62	52	75	49	42
35	63	44	53	28	21
83	64	44	36	34	22
92	65	43	53	59	39
11	66	39	43	43	22
15	67	34	31	26	23
28	68	32	33	42	21
27	69	31	14	12	23
25	70	30	41	38	26
34	71	27	31	30	17
77	72	27	36	23	17
60	73	25	23	35	32

80	74	24	28	22	13
19	75	24	32	31	23
47	76	19	22	19	15
64	77	19	22	18	15
89	78	18	21	21	11
58	79	17	28	22	16
84	80	17	24	18	8
66	81	15	21	19	12
67	82	15	19	13	9
38	83	15	29	26	16
68	84	15	20	14	11
39	85	15	43	34	11
30	86	15	21	27	16
9	87	15	22	18	13
48	88	15	22	16	10
55	89	15	32	25	24
37	90	15	19	17	13
13	91	15	21	18	11
29	92	15	15	13	10
21	93	15	26	23	16
57	94	15	16	15	7
24	95	15	13	10	10
63	96	15	15	11	7
